# Congenital abnormalities of the retinal vasculature in neurofibromatosis type I

**DOI:** 10.1038/s41598-020-69852-9

**Published:** 2020-07-30

**Authors:** Bo Young Chun, Jung Hyun Yoon, Byeong Jae Son, Su-Kyeong Hwang, Hyun Taek Lim

**Affiliations:** 1 Department of Ophthalmology, School of Medicine, Kyungpook National University, Daegu, South Korea; 2 Brain Science and Engineering Institute, School of Medicine, Kyungpook National University, Daegu, Korea; 3 Department of Pediatric Neurology, School of Medicine, Kyungpook National University, Daegu, Korea; 4 Department of Ophthalmology, Asan Medical Center, University of Ulsan College of Medicine, Seoul, Korea

**Keywords:** Anatomy, Biomarkers, Neurology, Signs and symptoms

## Abstract

The aim of this cross-sectional study was to investigate congenital abnormalities of the retinal vasculature (CARVs) in patients with neurofibromatosis type I (NF-1). Forty-eight patients (96 eyes) with NF-1 diagnosed according to the National Institutes of Health (NIH) criteria and 48 healthy controls were included in this study. Standard fundus photographs were obtained for each subject to evaluate the presence and frequency of CARVs. The sensitivity, specificity, and diagnostic accuracy of different cut-off numbers of CARVs were compared with those of the NIH criteria. Forty-four (91.7%) patients in the NF-1 group demonstrated either supranumeraty optic disc vessels or triple branching of the retinal vasculature, and 22 patients (45.8%) demonstrated both findings. The frequencies of these two CARVs were significantly different between the two groups (p < 0.00001). A cut-off value of either one for supranumerary optic disc vessels or triple branching showed the highest accuracy along with sensitivity and specificity of 91.7% and 87.5%. CARVs such as supranumerary optic disc vessels or triple branching were frequently observed in NF-1 patients, and their occurrence was unrelated to the age of patients. Thus, these CARVs could be added as new ophthalmologic manifestions for NF-1 and may potentially enable early diagnosis of NF-1.

## Introduction

Neurofibromatosis type-1 (NF-1) is one of the most common genetic disorders with an estimated incidence of 1 in 3,000 individuals, independent of ethnicity, race, and gender^1–3^. NF-1 is caused by inactivating mutations in the *NF1* gene, a tumor suppressor gene located on chromosome 17q11.2, which encodes the protein neurofibromin^3–5^. Since neurofibromin acts as a suppressor of the oncoprotein p21^Ras^ (Ras) and controls cell growth and survival, loss of neurofibromin leads to augmented cellular proliferation^5,6^. Therefore, individuals with NF-1 are vulnerable to developing low-grade tumors, such as neurofibromas and Lisch nodules; however, they are often undetectable in early childhood and do not reach maximum frequencies until adulthood^7–14^.

Early diagnosis and a multidisciplinary treatment approach are necessary for the management of patients with NF-1^10,11^. NF-1 is typically diagnosed on the basis of dermatologic findings and a positive family history of NF-1; however, the size and number of café-au-lait spots or skin-fold freckling may not meet the diagnostic criteria in the first years of life^7,11,12^. Since almost half of the patients with NF-1 have a negative family history due to its high spontaneous mutation rate, establishing an early diagnosis of NF-1 is even more difficult in very young patients with suspected NF-1^7,11,12^.

Nevertheless, NF-1 is more than a disease of tumor predisposition; it is a disorder of dysplasia^9^. The clinical features related to dysplasia in patients with NF-1 are vascular abnormalities and dysplastic bony lesions caused by dysfunction of *NF1* gene, which normally acts as a histiogenesis control gene^7,9,15,16^. Recently, loss of *NF1* gene expression in human endothelial cells has been reported to promote autonomous proliferation and abnormal vessel formation, and results in abnormal vascular structure^5,17^.

Based on the clinical rationale that establishment of an early diagnosis of NF-1 is necessary for patients with NF-1, and that these patients are predisposed to vascular abnormalities as well as tumor formation, the present study aims to evaluate the prevalence of congenital abnormalities of the retinal vasculature (CARVs) in patients with NF-1. We investigated the possibility of including the presence of CARVs as a new ophthalmic manifestation for NF-1, which may enable early diagnosis of NF-1.

## Results

The NF-1 group (48 patients) was composed of 16 male and 32 female patients with a mean age of 14 years (range; 3–35 years). Healthy controls (48 subjects) matched for age and sex with patients of the NF-1 group were assigned as the control group. There were no significant intergroup differences in the clinical characteristics such as best-corrected visual acuity (BCVA), refractive errors, intraocular pressure, and iris color (Table 1).

**Table 1:** Demographic and clinical characteristics of patients with neurofibromatosis type 1 and healthy controls.

Characteristics	Neurofibromatosis type 1 (n = 48)	Controls (n = 48)	P value
Age (years)	13.94 ± 6.92	14.02 ± 6.34	0.98^a^
Sex (Male:female)	16:32	16:32	1^b^
BCVA (Snellen chart)	20/20 (100%)	20/20 (100%)	1^a^
Refractive errors (diopters)	− 1.43 ± 1.76	− 1.32 ± 1.64	0.705^a^
Intraocular pressure (mmHg)	12.7 ± 2.2	12.5 ± 2.0	0.750^a^
Iris color	Dark brown (100%)	Dark brown (100%)	1^b^
Race	Asian (100%)	Asian (100%)	1^b^

The results are expressed as means ± SD (standard deviation) or as frequency (percentages).

*BCVA* best corrected visual acuity.

^a^p-values obtained using Student’s t-test for independent samples.

^b^p-value obtained using Fisher’s exact test.

Lisch nodules and optic gliomas were detected in 32 (67%) and three (6.3%) NF-1 patients, respectively, and none of the control participants. (Table 2). In the NF-1 group, there were statistically significant correlations between age and the presence of Lisch nodules (p = 0.037), and between age and the number of NIH diagnostic criteria (r = 0.369, p = 0.01).

**Table 2:** Frequency and diagnostic accuracy of National Institute of Health Diagnosic criteria of neurofibromatosis type 1.

	Neurofibromatosis type 1 (n = 48)	Controls (n = 48)	Accuracy
Café-au-lait macules	47/48 (98%)	1/48 (2%)	0.98
Freckling	26/48 (54.2%)	0/48 (0%)	0.77
Lisch nodules	32/48 (67%)	0/48 (0%)	0.83
Optic glioma	3/48 (6.3%)	0/48 (0%)	0.53
Neurofibromas	17/48 (35.4%)	0/48 (0%)	0.68
Distinctive osseous lesions	13/48 (27.1%)	NA	0.64
First-degree relative affected	25/48 (52.1%)	0/48 (0%)	0.76

*NA* not applicable.

**Figure 1:**
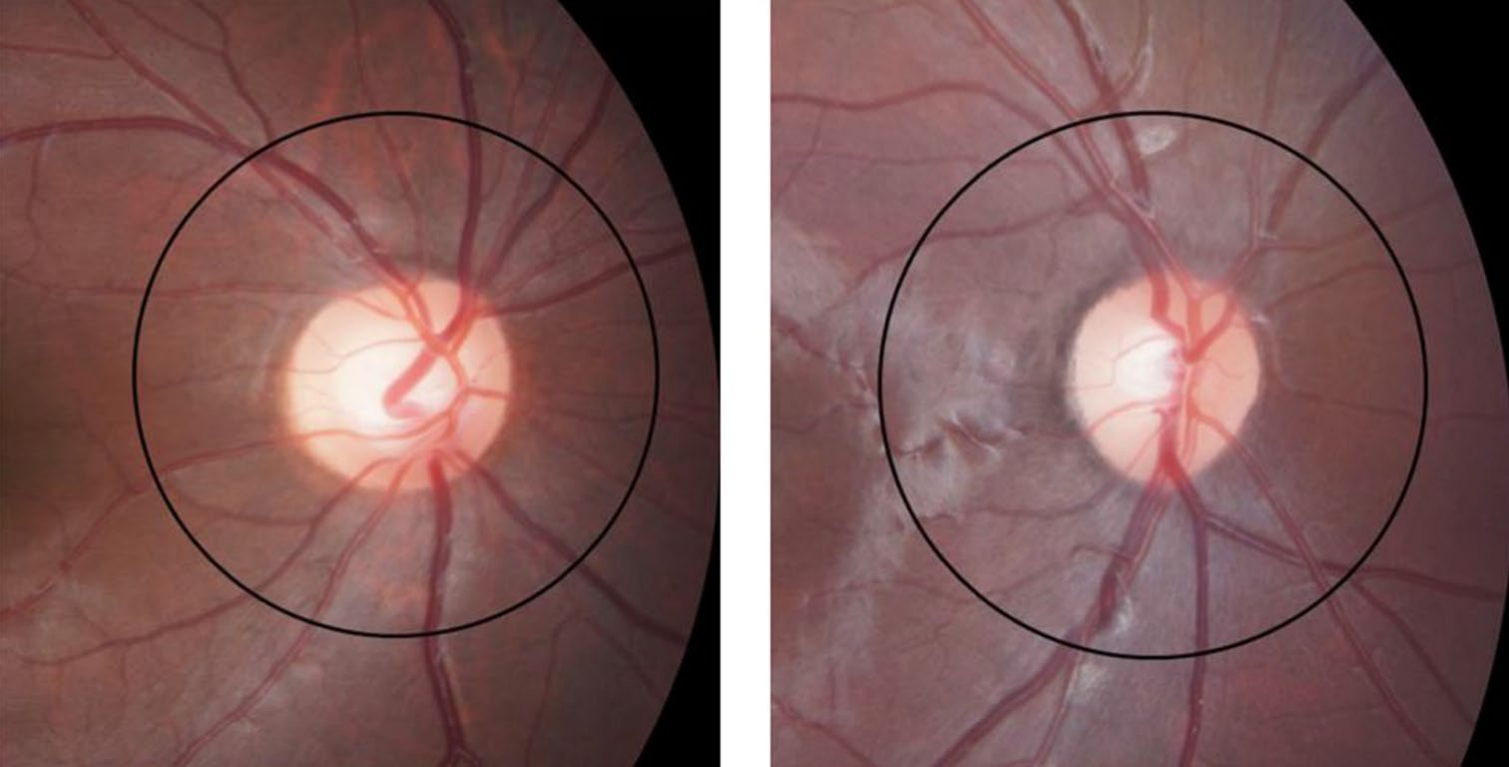
(Left) Supranumerary optic disc vessels observed in a patient with neurofibromatosis type 1, which is defined as having 18 or more branches of the central retinal vessels cross the optic nerve head margin that extends at least one disc diameter away. (Right) Normal optic disc vessels observed in a healthy control.

**Figure 2:**
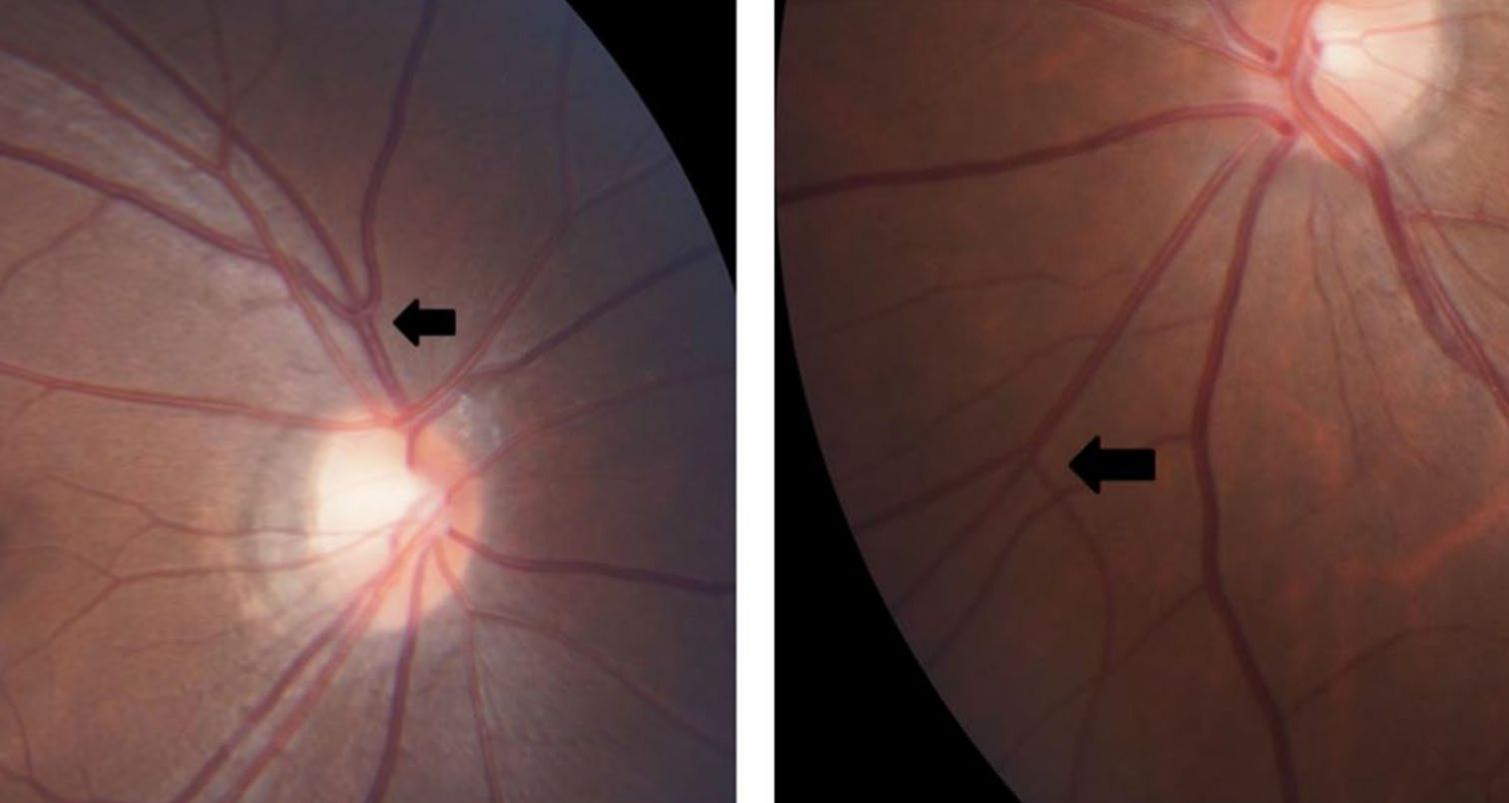
Triple branching of the retinal vasculature observed in patients with neurofibromatosis type 1, which defined as having a trifurcation of retinal vessel occurred at or near the optic disc, or further from the disc. (Left) Triple branching occurred near the optic disc. (Right) Triple branching occurred further from the disc.

In this study, most patients in the NF-1 group (44 patients, 91.7%) demonstrated either supranumerary optic disc vessels or triplc branching of the retinal vasculature (triple branching) detected by standard fundus camera photography (Table 3). Supranumerary optic disc vessels (Fig. 1), in which 18 or more branches of the central retinal vessels cross the optic nerve head margin that extends at least one disc diameter away^18^, were observed in 33 patients (68.8%) in the NF-1 group. Triple branching^19,20^ (Fig. 2) was observed in 33 patients (68.8%), and 22 patients (45.8%) demonstrated both supranumerary optic disc vessels and triple branching. In contrast, the control group contained five patients (10.4%) with supranumerary optic disc vessels and one patient (2.1%) with triple branching. There were statistically significant differences in the frequencies of supranumerary optic disc vessels (p < 0.00001) and triple branching between the NF-1 and control groups (p < 0.00001); both CARVs demonstrated a statistically significant intergroup difference in frequencies.

**Table 3:** Patients grouped by type and number of congenital abnormalities of the retinal vasculature observed in patients with neurofibromatosis type 1.

Threshold	Neurofibromatosis type 1	Controls
Supranumerary optic disc vessels		
0	15/48 (31.3%)	43/48 (89.6%)
≥ 1	33/48 (68.8%)	5/48 (10.4%)
≥ 2	22/48 (45.8%)	1/48 (2.1%)
Triple branching of the retinal vasculature		
0	15/48 (31.3%)	47/48 (97.9%)
≥ 1	33/48 (68.8%)	1/48 (2.1%)
≥ 2	9/48 (18.8%)	1/48 (2.1%)
Presence of supranumerary optic disc vessels or triple branching		
0	4/48 (8.3%)	42/48 (87.5%)
≥ 1	44/48 (91.7%)	6/48 (12.5%)
≥ 2	35/48 (72.9%)	1/48 (2.1%)
≥ 3	15/48 (31.3%)	0/48 (0%)
≥ 4	3/48 (6.3%)	0/48 (0%)

**Figure 3:**
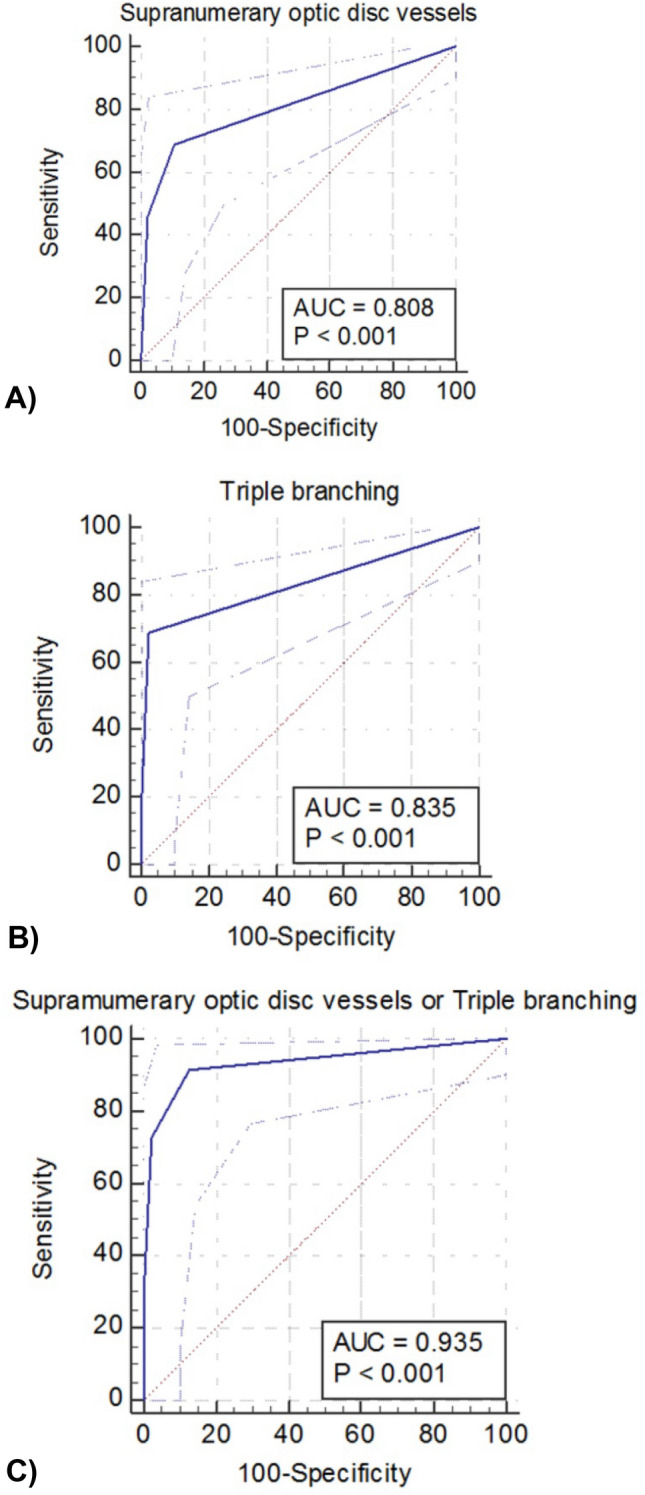
Receiver operating characteristic (ROC) curves at the cut-off value of 1 for supranumerary optic disc vessels (**A**), triple branching (**B**), either supranumerary optic disc vessels or triple branching (**C**) detected by standard fundus photography. In the presence of one or more supranumerary optic disc vessels, sensitivity and specificity were 68.8% and 89.4% with the area under the ROC curve (AUC) of 0.808 (95% confidence interval [CI] 0.714–0.881). In the presence of one or more triple branching, sensitivity and specificity were 68.8% and 97.9% with the AUC of 0.835 (95% CI 0.745–0.903). However, the highest accuracy was obtained with a cut-off value of one for either supranumerary optic disc vessels or triple branching detected by standard fundus photography. The sensitivity and specificity of standard fundus photography at this optimal cut-off value were 91.7% and 87.5%, respectively. The AUC was 0·935 (95% CI 0.865–0.975).

Using the number of CARVs as threshold values (Table 3), receiver operating characteristic (ROC) curves were constructed to determine the sensitivity and specificity of the CARVs as diagnostic indicators of NF-1. (Fig. 3A–C) Supranumerary optic disc vessels and triple branching demonstrated statistically positive results in the ROC analysis. In the presence of one or more supranumerary optic disc vessels, sensitivity and specificity were 68.8% and 89.4% with the area under the ROC curve (AUC) of 0.808 (95% confidence interval [CI] 0.714–0.881). In the presence of one or more triple branching, sensitivity and specificity were 68.8% and 97.9% with the AUC of 0.835 (95% CI 0.745–0.903). However, the highest accuracy was obtained with a cut-off value of one for either supranumerary optic disc vessels or triple branching detected by standard fundus photography. The sensitivity and specificity of this optimal cut-off value were 91.7% and 87.5%, respectively (Table 4). The AUC was 0.935 (95% Cl, 0.865–0.975).

**Table 4:** Sensitivity and specificity of cut-off numbers of congenital abnormalities of the retinal vasculature observed in patients with neurofibromatosis type 1.

	Sensitivity	Specificity
Number of supranumerary optic disc vessels		
1	68.75	89.6
2	45.83	97.9
Number of triple branching of the retinal vasculature		
1	68.75	97.9
2	18.75	100
Number of either presence of supranumerary optic disc vessels or triple branching		
1	91.67	87.5
2	72.92	97.9
3	31.25	100
4	0	100

There was no significant correlation between age and the frequency of these two CARVs in the NF-1 group (r = 0.045, p = 0.763). While 29 (60.4%) NF-1 patients demonstrated both Lisch nodules and CARVs, 15 (31.3%) patients showed only CARVs, but no Lisch nodules, three (6.3%) NF-1 patients showed only Lisch nodules without CARVs, and one patient (2.1%) showed neither Lisch nodules nor CARVs.

The diagnostic accuracy of the presence of supranumerary optic disc vessels was 0.79 and that of triple branching was 0.83. For assessments based on the presence of either supranumerary optic disc vessels or triple branching detected by standard fundus photography, the diagnosic accuracy was 0.90, which was slightly lower than that of café-au-lait spots (0.98) but higher than those of the other NIH diagnostic criteria, including Lisch nodules (0.83). The number of supranumerary optic disc vessels and the presense of triple branching demonstrated excellent intraobserver (κ = 0.956 and κ = 0.912, respectively) and interobserver (κ = 0.915 and κ = 0.870, respectively) agreements.

## Discussion

In the current study, we observed that the majority (44 of 48 patients, 91.7%) of NF-1 patients demonstrated either supranumerary optic disc vessels or triple branching detectable by standard fundus photography. Particularly, the frequencies of these two CARVs were significantly higher in the NF-1 group than in the control group. (p < 0.00001) Although there was a significant correlation between the age and the number of NIH diagnostic criteria, the presence of CARVs did not appear to be related to the age of the NF-1 patients. The diagnostic accuracy of the presence of either suprranumerary optic disc vessels or triple branching was 0.90, which was slightly lower than the accuracy for café-au-lait spots (0.98), but higher than those for the other NIH diagnostic criteria, including Lisch nodules (0.83) in the NF-1 group.

There was a significant correlation between age and the number of NIH diagnostic criteria in this study, and our results were consistent with previous studies in which the clinical features of NF-1 became more evident with age^7,11,12,21,22^. Neurofibromas appear in mid- to late childhood and are present in most adult patients with NF-1, and Lisch nodules are rare in small children but occur in most NF-1 patients older than ten years^7,11,13^. Viola et al.^21^ reported that the frequency of Lisch nodules was 43% in pediatric NF-1 subjects and 72% in NF-1 subjects of all ages. Similar results were reported by Vagge et al.^22^^,^ who showed that the frequency of Lisch nodules was 48.7% in the pediatric population of NF-1 patients. Therefore, many children who are proven to have NF-1 later do not meet the NIH diagnostic criteria in the first years of life^7,12^. The reliability of NIH diagnostic criteria has been reported to improve every year as the child grows older since the frequencies of other clinical signs of NF-1 increase rapidly during childhood^7,11^.

In contrast to the NIH diagnostic criteria, the CARVs observed in this study were not age-dependent in the NF-1 group. We assume that these findings are congenital in origin and non-progressive in nature owing to their pathophysiology, i.e., embryonic dysplasia caused by the loss of *NF1* gene function^9^. In addition, the diagnostic accuracy for the presence of suprranumerary optic disc vessels or triple branching was only slightly lower than that for café-au-lait spots and higher than those for the other NIH diagnostic criteria, in the NF-1 group. Thus, in very young children with suspected NF-1, especially those with a negative family history, the presence of the CARVs observed in this study can be more helpful for diagnosis of NF-1 than other clinical signs with progressive characteristics, such as Lisch nodules or neurofibromas. In the current study, the highest diagnostic accuracy was obtained at the cut-off value of one for either supranumerary optic disc vessels or triple branching. The sensitivity and specificity of this optimal cut-off value were 91.7% and 87.5%, respectively. Our results are comparable to those reported by Viola et al.^21^^,^ who showed that the sensitivity and specificity of near-infrared reflectance (NIR) imaging at the optimal cut-off value of 1.5 choroidal nodules were 83% and 96%, respectively. The AUC was 0.935 in this study, which was slightly superior than the value (0.904) reported by Viola et al.^21^.

Recently, choroidal abnormalities or choroidal nodules detected by NIR imaging with or without idocyanine-green fundus angiography have been suggested to be a new diagnostic criterion with an estimated prevalence between 60 and 100% in patients with NF-1^21–25^. However, these choroidal abnormalities are undetectable using conventional ophthalmoscopic examination; they can only be detected by NIR with a scanning laser ophthalmoscope^21–25^. In addition, the extent of choroidal involvement is variable and its prevalence increases with age, since choroidal nodules consist of abnormally proliferating Schwann cells^21,26^. Therefore, in very young children with suspected NF-1, the presence of choroidal nodules detected by NIR imaging is technically difficult to examine and may be underestimated like most clinical features of NF-1, which demonstrate a progressive nature and extreme variability.

Moreover, retinal microvascular abnormalities in patients with NF-1 have been reported in the literature^27–29^. Muci-Mendoza et al.^27^ reported that retinal microvasculature abnormalities such as corkscrew retinal vessels detected by fluorescein angiogram were observed in 12 of 32 (37.5%) patients with NF-1. Parrozzani et al.^28^ reported that retinal microvascular abnormalities that appeared as small, tortuous vessels with a spiral/corkscrew aspect and detected by infrared confocal scanning laser ophthalmoscopy images were present in 18 of the 294 (6.1%) patients with NF-1. The presence of retinal microvascular abnormalities did not correlate with age and sex and not with any specific features of NF-1, including Lisch nodules and choroidal abnormalities^28^. Although those findings were present in none of the healthy subjects, the prevalence of retinal microvascular abnormalities was too low (6.1%) to be regarded as a new diagnostic criterion in patients with NF-1^28^. Recently, Moramarco et al.^29^ identified retinal microvascular abnormalities detected by NIR optical coherence tomography in 31.4% of 334 patients with NF-1. The simple vascular tortuosity was the most frequently observed type among microvascular alterations, and positively associated with age and the presence of the neurobibromas^29^.

We assume that the pathogenesis of CARVs observed in this study results from interference of the normal structural development of the retinal vasculature in the fetal human eye induced by mutations in the *NF1* gene, which normally acts as a histiogenesis control gene^9,18,19,30^. In normally developing human eye, the fetal vasculature of vitreous including the hyaloid vasculature initially develops around 4–6 weeks of gestation (WG) and expands until 12 WG^30^. This transient fetal vasculature remains until a retinal vasculature forms and then regresses by apoptosis with the assistance of macrophages and hyalocytes^30,31^. The emergence of the initial retinal vasculature as angioblasts in linear arrangement in the peripapillary region, as well as the expression of the vascular endothelial growth factor (VEGF) in angioblasts and endothelial cells can be shown approximately at 12–14 WG^30,32^. The proliferation of the retinal vasculature is observed at 18 WG; then, the superficial retinal vasculature expands by vasculogenesis at 21 WG, and the deep retinal capillary network forms by angiogenesis at 25–26 WG^30,33,34^. Neurofibromin is normally expressed in endothelial and smooth muscle cells of blood vessels, and it has been suggested that NF-1 related vasculopathy results from neurofibromin deficiency in these cells^9, 15,16^. Recent studies reported that neurofibromin tightly controls endothelial cell proliferation; neurofibromin deficiency activates Ras-dependent kinases, and enhances endothelial cell proliferation^5,17^. Moreover, neurofibromin deficiency alters vascular morphogenesis in developing vascular structures^17^. Therefore, we infer that triple branching detected in patients with NF-1 can be explained by hyperplastic morphogenesis in response to activated Ras-related signaling by neurofibromin deficiency during fetal development of the retinal vasculature^5,17,30,34^. In in vitro experiment, the loss of *NF1* gene resulted in the appearance of abnormal vascular structures such as triple branching^17^. We postulate that supranumerary optic disc vessels observed in patients with NF-1 may be induced by the apoptosis failure of fetal hyaloid vessels around the optic disc or by uncontrolled vasculogenesis of the superficial retinal vasculature during fetal development, since the *NF1* gene normally acts as a histiogenesis control gene^9,30,34^.

Our results are limited by the fact that we did not investigate an association between the presence of CARVs and systemic vascular abnormalities in patients with NF-1. It is reported that vasculopathy is over seven times more likely to occur in patients with NF-1 under 30 years compared to their unaffected peers^7,35^. In addition, one of major causes of increased mortality among patients with NF-1 is systemic vasculopathy, particularly cerebrovascular disease^9,15,16^. Our future study will focus on investigation of the possible correlation between CARVs and cerebrovascular abnormalities. If there is any correlation between these two factors, an early diagnosis of systemic vascular abnormalities may decrease the mortality from unrecognized vasculopathy in patients with NF-1. Finally, supranumerary optic disc vessels were also observed in patient with Down syndrome^19,36^. Several studies reported that 40 to 54.5% of patients with Down syndrome showed 18 or more vessels crossing the disc margin while 5 to 6% of controls displayed it^19,36^. Parsa and Almer suggested that supranumerary optic disc vessels may indicate reduced systemic angiogenesis in Down syndrome^37^. They postulated that a mild deficiency of angiogenesis inhibited central retinal vessel formation, and multiple cilioretinal vessels become more prominent^37^. However, there is no in vitro experimental data on their theory. Nevertheless, it is inferred that supranumerary optic disc vessels may be one of clinical manifestations induced by abnormal angiogenesis during fetal development of the retinal vasculature.

In conclusion, the present study offers the first data on the prevalence of CARVs in the NF-1 population. We observed that the majority (44 of 48 patients, 91.7%) of NF-1 patients demonstrated either supranumerary optic disc vessels or triple branching by standard fundus photography. Particularly, the frequencies of these two CARVs were significantly higher in the NF-1 group than in the control group. Detection of these CARVs can be easily accomplished by a non-invasive method with standard fundus photography, or even by routine fundus examinations. In addition, the presence of CARVs observed in this study is irrelevant to the age of patients with NF-1. Therefore, in very young children with suspected NF-1, especially with a negative family history, the presence of CARVs may potentially enable early diagnosis of NF-1. Authors suggest that CARVs such as supranumerary optic disc vessels or triple branching could be added as new ophthalmologic manifestions for NF-1.

## Patients and methods

This study was conducted between March 2011 and May 2019 at the Department of Ophthalmology of the Kyungpook National University Hospital. The protocol of this study was approved by the Institutional Review Board of Kyungpook National University Hospital and was conducted in accordance with the tenets of the Declaration of Helsinki. All patients and controls (or their parents or guardians if they were children) gave their informed consent.

Patients diagnosed with NF-1 on the basis of the stringent National Institutes of Health (NIH) criteria were examined. At least two of the following criteria are required for the diagnosis of NF-1; six or more café-au-lait spots, skin-fold freckling, two or more cutaneous neurofibromas, one plexiform neurofibroma, distinctive bone lesions, optic pathway glioma, two or more Lisch nodules, and a first-degree relative with NF-1^38^. We retrospectively analyzed the fundus photogtaphs from patients who were enrolled in our ongoing patient registry. Healthy age- and sex-matched controls were recruited from patients of the ophthalmology unit who presented for routine ophthalmological examination. Patients who did not undergo fundus examinations due to poor cooperation or media opacity, a history of ocular trauma or surgery, and any other ocular or systemic disease were excluded.

All subjects underwent a comprehensive ophthalmologic examination, including measurements of BCVA, refractive errors, and intraocular pressure, slit-lamp examination with assessment of Lisch nodules, indirect binocular fundus ophthalmoscopy, and photographic recording of fundus images with the VISUCAM 224 fundus camera (Carl Zeiss Meditec AG) in pupillary mydriasis. Data regarding the patient’s age at the diagnosis of NF-1, sex, BCVA, refractive errors, and fundus findings were collected. Detailed analysis of fundus photographs and comparisons with those of normal controls were performed. Two investigators who were masked to the patient’s information independently examined fundus photographs. In case of disagreement between the two investigators, the final decision was made by consulting a third investigator.

### Definition of congenital abnormalities of the retinal vasculature (CARVs)

Supranumerary optic disc vessels was defined as having 18 or more branches of the central retinal vessels cross the optic nerve head margin that extends at least one disc diameter away^19^ (Fig. 1). Triple branching of the retinal vasculature was defined as having a trifurcation of retinal vessel occurred at or near the optic disc, or further from the disc^20,21^ (Fig. 2).

### Statistical analysis

The presence of CARVs was recorded using descriptive methods. Frequencies of each CARV detected by fundus photography were compared between NF-1 patients and normal controls by Fisher’s exact test. Intraobserver and interobserver agreement over fundus photographs was calculated using Cohen’s kappa statistics, where agreement was defined according to previously known guidelines^39,40^. Pearson’s correlation coefficients were calculated between age and the total number of NIH diagnostic criteria in patients with NF-1. Pearson’s correlation coefficients were also calculated between age and the total number of CARVs in patients with NF-1. ROC curve was constructed to demonstrate the variability of sensitivity and specificity for each and the total number of CARVs detected by fundus photography. The AUC was selected as the global index of diagnostic accuracy. The diagnostic accuracy of each CARV was compared with those of the NIH diagnostic criteria. P values of 0.05 or less were considered statistically significant.
